# Reliable Establishment of Human Sarcoma Xenografts in the Nude Rat

**DOI:** 10.1080/13577149977767

**Published:** 1999-09

**Authors:** Peggy T. Tinkey, Mira Milas, Raphael E. Pollock

**Affiliations:** 1 Department of Veterinary Medicine and Surgery The University of Texas M. D. Anderson Cancer Center 1515 Holcombe Blvd. Houston TX 77030 USA; 2 Department of Surgical Oncology The University of Texas M. D. Anderson Cancer Center 1515 Holcombe Blvd. Houston TX 77030 USA

## Abstract

*Purpose.* The ability to establish consistent human tumor xenografts in experimental animals is a crucial part of preclinical investigations.The goal of this study was to develop a method of establishing a human tumor xenograft in the leg of a nude rat for evaluation of new surgical and molecular methods of treatments of human extremity sarcoma.

*Methods and results.* Initial attempts to produce sarcoma nodules by subcutaneous injection of a human leiomyosarcoma tumor cell suspension (SKLMS-1) resulted in tumor nodule formation in only four of 10 sites (40%).The xenograft method was modified to include younger nude rats of a different source and substrain (HSD:rnu/rnu, 5–9 weeks old), treated with 500 cGy whole-body irradiation, and the transplantation of tumor cells or small tumor fragments which had been embedded in Matrigel.These changes improved the tumor take rate per site to 52/52 (100%).Tumor nodules demonstrated rapid and progressive growth and histological features consistent with the original human sarcoma.

*Discussion.* Successful human leiomyosarcoma establishment in these nude rats permits the investigation of sarcoma biology and treatment with surgical procedures for which a mouse model would be inadequate. In this study we identified modifications in technique which enhanced the xenografting of a leiomyosarcoma cell line in nude rats; these techniques may increase tumor take rates for other tumor types as well.


*Sarcoma* (1999) **3**, 129–133

SHORT REPORT

### Reliable establishment of human sarcoma xenografts in the nude rat

PEGGY T. TINKEY,[1] MIRA MILAS[2] & RAPHAEL E. POLLOCK[2]

*Departments of [1]Veterinary Medicine and Surgery and [2]Department of Surgical Oncology, The University of Texas M. D. Anderson Cancer Center, 1515 Holcombe Blvd., Houston, TX 77030, USA*

**Key words:** *tumor xenograft, nude rat, sarcoma*

**Abstract**

*Purpose.* The ability to establish consistent human tumor xenografts in experimental animals is a crucial part of preclinical investigations. The goal of this study was to develop a method of establishing a human tumor xenograft in the leg of a nude rat for evaluation of new surgical and molecular methods of treatments of human extremity sarcoma.

*Methods and results.* Initial attempts to produce sarcoma nodules by subcutaneous injection of a human leiomyosarcoma tumor cell suspension (SKLMS-1) resulted in tumor nodule formation in only four of 10 sites (40%). The xenograft method was modified to include younger nude rats of a different source and substrain (HSD:rnu/rnu, 5–9 weeks old), treated with 500 cGy whole-body irradiation, and the transplantation of tumor cells or small tumor fragments which had been embedded in Matrigel. These changes improved the tumor take rate per site to 52/52 (100%). Tumor nodules demonstrated rapid and progressive growth and histological features consistent with the original human sarcoma.

*Discussion.* Successful human leiomyosarcoma establishment in these nude rats permits the investigation of sarcoma biology and treatment with surgical procedures for which a mouse model would be inadequate. In this study we identified modifications in technique which enhanced the xenografting of a leiomyosarcoma cell line in nude rats; these techniques may increase tumor take rates for other tumor types as well.

### Introduction

Human soft tissue sarcomas are a rare group of heterogeneous tumors characterized by aggressive local growth and hematogenous metastases, most often to the lungs. Surgical resection, with or without radiotherapy, is the cornerstone of the local treatment of these sarcomas. Surgical methods that allow limb preservation for patients who would otherwise require amputation have also been developed.[1,2] An example is isolated limb perfusion (ILP), which is used to deliver high doses of chemotherapeutic agents to an extremity tumor whose vascularity has been temporarily isolated from the remainder of the body, thereby preventing patient exposure to systemically toxic drug concentrations.[2] Despite such advances, the overall dismal prognosis for sarcoma patients and the need to improve current treatment options necessitate an active research effort and reliable preclinical animal models.[3]

Orthotopic xenografts of human tumors in nude mice are an accepted model for in vivo biological and preclinical studies;[4] however the small size of mice limits their usefulness in many surgical applications. The investigation of sarcoma tumor biology and surgical techniques, such as ILP, in a larger laboratory animal therefore requires that a consistently producible human tumor xenograft model be achievable in such an animal.

We report here a method of generating human sarcomas in the leg or flank of the nude rat that results in excellent tumor take rates and consistent, reliable growth patterns. The principles of xenograft establishment described here for sarcomas may be useful for research applications involving other types of human tumors.

### Methods

### *Animals*

Female Rowett nude rats, 4–6 weeks old, were obtained from the National Cancer Institute (Frederick Cancer Research Facility, Frederick, MD; strain Cr:NIH-RNU) or from Harlan Sprague–Dawley (Indianapolis, IN; strain HSD:rnu/rnu). The animals were housed three per cage in sterilized rat microisolator cages (Lab Products, Seaford, DE) containing corncob bedding (Bed'o'Cob; Northeastern Products Corp., Caspain, MO). The temperature was

Correspondence to: Raphael E. Pollock, The University of Texas M. D. Anderson Cancer Center, Department of Surgical Oncology, 1515 Holcombe Blvd., Box 106, Houston, TX 77030, USA. Tel: +1 713 792 6928; Fax: +1 713 792 0722; e-mail: rpollock@mdanderson.org

1357-714X print/1369-1643 online/99/020129-05 © 1999 Taylor & Francis Ltd maintained at 22±2°C and humidity at 50±10%. Animals were provided a 12-h light–dark cycle and *ad libitum* autoclaved food (Harlan-Teklad, Madison, WI) and water throughout the study. The experiments were approved by the Institutional Animal Care and Use Committee at The University of Texas M.D. Anderson Cancer Center. Animals received humane care in accordance with the Animal Welfare Act and the NIH *Guide for the Care and Use of Laboratory Animals*.

### *Tumors*

The human leiomyosarcoma SKLMS-1 was obtained from the American Type Culture Collection (Rockville, MD). SKLMS-1 is a well-characterized human sarcoma cell line and contains a known p53 tumor suppressor gene mutation.[5,6] Tumor cells were either maintained in cell culture or passaged in nude rat subcutaneous tissue. Cells in culture were maintained in DMEM/F12 medium containing 10% fetal bovine serum (FBS, Life Technologies, Inc., Gaithersburg, MD) in a 37°C humidified incubator containing 5% $CO_2$ in air. Cells were subcultured 1:3 by trypsinization upon reaching 90% confluence. For experiments, 90% confluent cells were harvested by trypsinization, washed in phosphate-buffered saline (PBS), and assessed for viability by trypan blue dye exclusion. The cells were then pelleted and resuspended in either PBS or Matrigel (Becton-Dickinson, Bedford, MA). For passaged tumor, subcutaneously growing SKLMS-1 nodules were resected aseptically and trimmed of necrotic tissue. The remaining viable tumor was rinsed in cold PBS, cut into 3 × 2-mm slivers, immersed in Matrigel, and immediately implanted into recipient rats. Matrigel is a solubilized basement membrane preparation originally extracted from a mouse sarcoma; it contains extracellular matrix proteins, such as laminin and collagen, and growth factors.[7,8]

### *Radiation*

Nude rats received a single dose of 500 cGy total-body irradiation which was administered using a cobalt-60 source (Eldorado-8 teletherapy machine, Atomic Energy of Canada, Ltd.) delivering approximately 57 cGy/min. During irradiation, awake rats were placed in a mouse microisolator cage and covered with a ¼-inch solid Lucite platform constructed to fit the inner cage dimensions and minimally contact the dorsal surface of the rats. The rats were used in tumor implantation experiments 4 days after irradiation.

### *Tumor implantation*

Rats were anesthetized using Metophane (Mallinckrodt Veterinary, Mundelein, IL) in an inhalation anesthetic chamber. The skin over the implantation site was wiped clean with 70% ethanol. For injection, 200 μl of SKLMS-1 cell suspension containing $10 \times 10^6$ cells was inoculated into the subcutaneous tissue in the rat flank or hind leg using a tuberculin syringe with a 28-Ga needle (Becton-Dickinson). For implantation, a 5-mm incision was made in the skin overlying the flank or leg, and a single 3 × 2-mm sliver of tumor tissue was delivered through the incision site using a trocar and deposited 1 cm away in the subcutaneous tissue. The skin was closed using a single wound clip (Becton-Dickinson).

### Results

The goal of these experiments was to establish a method of tumor implantation that would lead to consistent and reliable human sarcoma xenografts in the rat flank and leg. The results of the experiments are summarized in Table 1. The initial experiments were conducted using eight NCI nude rats which were either 9 weeks old ($n$=5) or 12 weeks old ($n$=3). All rats were subcutaneously inoculated at each injection site with a 200-μl suspension of $10 \times 10^6$

**Table 1**. *Tumor takes rates achieved with various modifications in xenotransplantion method*

| Rat strain and vendor | Quantity and age of rats | TBI | Tumor cell preparation | Inoculation site | Tumor take/site (%) at 4 weeks PI |
|---|---|---|---|---|---|
| Cr:NIH-RNU; NCI | $n$=5; 9 weeks | No | Cell suspension | 1 flank and 1 leg/rat | Leg 1/5 (20%)<br>Flank 3/5 (60%)<br>All sites 4/10 (40%) |
| Cr:NIH-RNU; NCI | $n$=3; 12 weeks | No | Cell suspension | 1 flank and 1 leg/rat | Leg 0/3 (0%)<br>Flank 0/3 (0%)<br>All sites 0/6 (0%) |
| HSD:rnu/rnu; HSD | $n$=10; 7 weeks | 500 rad | Tumor sliver + Matrigel | $n$=6: 2 flanks and 2 legs/rat<br>$n$=4: 1 flank and 2 legs/rat | Leg 20/20 (100%)<br>Flank 16/16 (100%)<br>All sites 36/36 (100%) |
| HSD:rnu/rnu; HSD | $n$=6; 12 weeks | 500 rad | Tumor sliver + Matrigel | 2 legs/rat | Leg 12/12 (100%) |
| HSD:rnu/rnu; HSD | $n$=2; 9 weeks | 500 rad | Cell suspension + Matrigel | 2 flanks/rat | Flank 4/4 (100%) |

PI, post-implantation.

SKLMS-1 sarcoma cells in PBS. There were two injection sites per rat, the flank and hind limb, and these were inspected daily by visual examination and palpation for evidence of tumor growth. In the group of rats inoculated at 9 weeks of age, one rat developed a small tumor nodule (5 mm) in the leg 2 weeks post-injection; no rat had evidence of tumor growth in the flank. At 3 weeks post-injection, two rats had tumor nodules in the leg and another two in the flank. At both 4 and 12 weeks post-injection, only one rat retained a growing tumor nodule in the leg; the nodule in the other rat had spontaneously regressed. Three rats had tumor nodules in the flank. Maximum tumor nodule diameter at 12 weeks was 1.5 cm. At no time did any of the three rats inoculated at 12 weeks of age exhibit tumor growth at any site. For the younger rats, therefore, the overall tumor take rate for all sites by 12 weeks was four of 10 (40%) and respective values for the leg and flank specifically were one of five (20%) and three of five (60%).

The tumor take rates observed with injection of SKLMS-1 cells in PBS were unsuitable for studies that require many rats and timely development of tumor nodules. To optimize the success of xenografting these sarcoma cells, we modified the above implantation procedure by using younger rats (5–7 weeks old) from a different supplier (Harlan Sprague–Dawley), treating them with 500 cGy total-body irradiation to achieve further immunosuppression, adding Matrigel to the tumor, and transplanting tumor fragments harvested from a donor rat. Although each of these changes individually elicited some improvement (data not shown), the best results were achieved when all modifications were applied.

Four days after irradiating a group of 10 7-week-old rats, SKLMS-1 tumor in 3 × 2-mm slivers coated with Matrigel was implanted subcutaneously in either three sites or four sites per rat to yield 36 total inoculation sites. Six rats were inoculated in both flanks and both legs; four rats were inoculated in one flank and both legs. No radiation-related adverse effects, such as skin changes or lethargy, were noted. Eight days after implantation, tumor nodules were easily palpable at all sites, and all grew to a diameter of 1 cm by 2 weeks post-implantation (Fig. 1). The tumor take rate per site with this method was 36/36 (100%). A similar time course of tumor growth and the same xenotransplantation rate was achieved in slightly older rats who were also irradiated at 12 weeks of age. In this group of rats, tumor nodule formation occurred at 12/12 sites (100%). Furthermore, two Harlan Sprague–Dawley rats were irradiated at 7–9 weeks of age and injected subcutaneously in both flanks with SKLMS-1 cells suspended in Matrigel (200 μl containing $10 \times 10^6$ cells). Approximately 5 weeks after injection, 1-cm nodules were palpable at all four sites and exhibited continued growth.

### Discussion

The use of human xenografts in laboratory animals is an essential component of preclinical cancer therapy investigations. Xenografts of human tumors are most commonly modeled in nude mice,[4] whose small size is convenient for most experimental applications but limits the investigation of more complex surgical procedures. For these kinds of studies, the nude rat is more appropriate. The nude rat has an autosomal recessive mutation which results in the lack of a functional thymus.[9] T lymphocytes and cell-mediated immunity are markedly reduced, and xenograft rejection is diminished or delayed.[9] Because of their larger size, nude rats permit easier performance of surgical manipulations, especially on small structures such as blood vessels. The primary disadvantage of the nude rats is their relative immune competence compared to athymic nude mice. Many human tumor xenografts in nude rats show either greatly diminished tumor take rates compared to those in nude mice or spontaneous regression of the tumors.[10–16] Selected interventions, such as whole-body irradiation, have been reported to improve tumor take rates for human lung cancer models in nude rats.[17,18]

The purpose of these experiments was to identify components of tumor implantation technique that would lead to consistent and reliable human sarcoma xenografts in the rat flank and leg, sites important for sarcoma research applications. Particularly necessary was the establishment of similarly sized tumor nodules growing at a predictable rate in rat limbs intended to undergo ILP with biochemotherapeutic agents. In the process of trying to establish this xenograft method, we observed highly variable tumor take rates and tumor growth properties. Such variability does not seem to affect syngeneic rat sarcoma transplants, which lead to tumor establishment within about 1 week.[19,20] A method of direct tumor implantation used with a syngeneic rat fibrosarcoma for successful ILP studies with genetic therapy[21] did not result in consistent tumor take rates when applied to the human SKLMS-1 leiomyosarcoma tumor. This circumstance allowed us to identify several factors that appear to be important in determining xenograft tumor take rates. These include the vendor source, strain and age of rat, presence or absence of whole-body irradiation, form of transplanted tumor cells (solid fragment of tumor tissue versus cell suspension), and presence or absence of a xenograft environment enriched with growth factors.

The need for these modifcations reflects varying degrees of immune competency of nude rats, the malignant phenotype of the particular tumor, and the importance of local tumor growth factors in establishing xenografts. The immunological characteristics of nude rats may vary depending on the rats' source and strain.[9] Maximum immunosuppression may require changing rat strains or vendor sources, using younger rats (≤11 weeks old), and

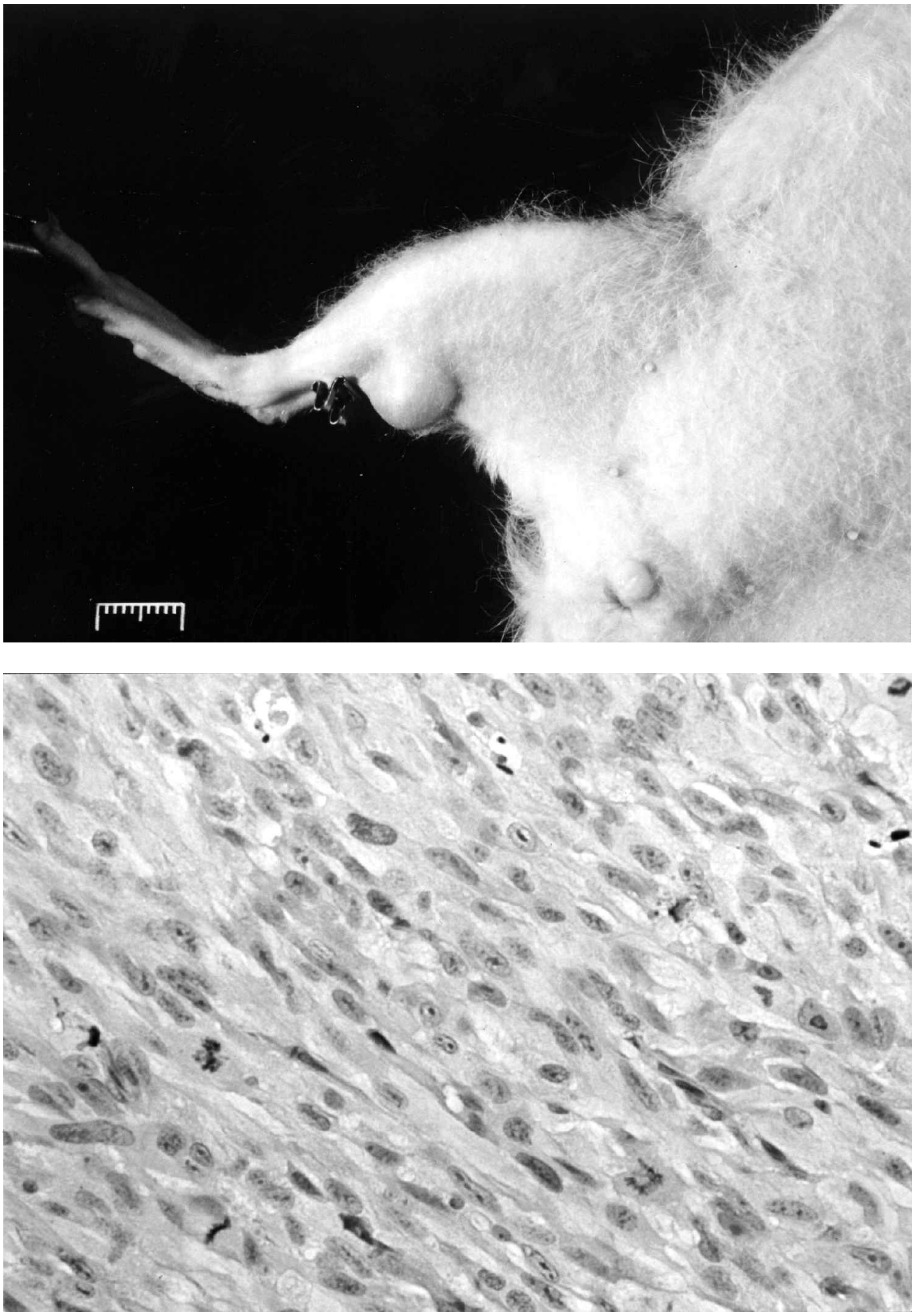

**Fig. 1. (A)** *Nude rat with a human leiomyosarcoma nodule growing in the medial aspect of its lower limb. The rat underwent 500 cGy total-body irradiation 4 days prior to implantation of a 3 × 2-mm fragment of SKLMS-1 tumor embedded in Matrigel. The tumor reached 1 cm in size by 2 weeks, and consisted of viable heterogeneous cells with appearance consistent with human leiomyosarcomas* **(b)**.

further lymphocyte depletion of the selected rats through whole-body irradiation. The inherent aggressiveness of a human tumor may dictate how many of these modifications are needed to ensure xenograft establishment. Total-body irradiation alone was sufficient in some lung cancer models.[17,18,20] Manipulation of the xenograft environment may also improve tumor take rates. Implantation of an established tumor fragment harvested from a donor rat may enhance intercellular interactions in a new host, compared to inoculation of a loose suspension of cultured tumor cells.[22,23] The cellular environment may be further enhanced by exogenous growth factors. Matrigel is a solubilized basement membrane preparation. We found that the administration of the tumor fragments in conjunction with Matrigel, which contains extracellular matrix proteins and growth factors,[7,8] resulted in optimum tumor take rates and growth pattern consistency in our sarcoma model.

In summary, we have reported a method of producing human sarcoma xenografts in the leg of the nude rat, using a human leiomyosarcoma cell line, which yields excellent tumor take rates and consistent, reliable growth patterns. The use of young nude rats of a select strain, combined with the administration of 500 cGy whole-body irradiation and the use of Matrigel-soaked tumor implants, resulted in high tumor take rates with no tumor nodule regression over time. This makes it possible to continue the investigation of such therapeutic agents as chemotherapy drugs or gene therapy constructs, and also brings to attention aspects of xenografting technique that may be modified to improve tumor take rates of other human tumor xenografts in nude rats.

### Acknowledgement

This work was partially supported by NIH R01 CA67802 (to R.E.P) and the M.D. Anderson Cancer Center NIH Core Grant CA16672. M.M. is supported by NIH T32 Training Grant CA09559 (to R.E.P.)

### References

1 Pisters PW, Fein DA, Somlo G. Soft-tissue sarcomas. In: Pazdur R, *et al*., eds. *Cancer management: a multidisciplinary approach*. First Ed. Huntington, NY: PRR, 1996:491–505.

2 Eggermont AM, Schrafford T, Koops H, *et al*. Isolated limb perfusion with tumor necrosis factor and melphalan for limb salvage in 186 patients with locally advanced soft tissue extremity sarcomas. The cummulative multicenter European experience. *Ann Surg* 1996; 244:756–64.

3 Milas M, Yu D, Pollock RE. Advances in the understanding of human soft tissue sarcomas: molecular biology and therapeutic strategies. *Oncol Rep* 1998; 5:1275–9.

4 Fidler IJ. Rationale and methods for the use of nude mice to study the biology and therapy of human cancer metastasis. *Cancer Metastasis Rev* 1986; 5:29–49.

5 Stratton MR, Moss S, Warren W, *et al*. Mutation of the p53 gene in human soft tissue sarcomas: Association with abnormalities of the RB1 gene. *Oncogene* 1990; 5:1297–301.

6 Pollock RE, Lang A, Ge T, *et al*. Wild-type p53 and a p53 temperature-sensitive mutant suppress human soft tissue sarcoma by enhancing cell cycle control. *Clin Cancer Res* 1998; 4:1985–94.

7 Nicosia R, Ottinetti A. Modulation of microvascular growth and morphogenesis by reconstituted basement membrane gel in three-dimensional cultures of rat aorta: a comparative study of angiogenesis in Matrigel, collagen, fibrin, and plasma clot. *In Vitro Cell Dev Biol* 1990; 26:119–28.

8 McGuire PG, Seeds NW. The interaction of plasminogen activator with a reconstituted basement membrane matrix and extracellular macromolecules produced by cultured epithelial cells. *J Cell Biochem* 1989; 40:215–27.

9 Quimby FW, Bosma MJ. Hereditary immunodeficiencies. In: National Academy of Sciences, ed. *Immunodeficient rodents: a guide to their immunobiology, husbandry, and use*. 1st Ed. Washington, DC: National Academy Press, 1989: 125–8.

10 Festing MFW, May D, Connors TA, *et al*. An athymic nude mutation in the rat. *Nature* 1978; 274:365–6.

11 Colston MJ, Fieldsteel AH, Dawson PJ. Growth and regression of human tumor cell lines in congenitally athymic (*rnu/rnu*) rats. *J Nat Cancer Inst* 1981; 66:843–8.

12 Drewinko B, Moskwa P, Lotzova E, Trujillo JM. Successful heterotransplantation of human colon cancer cells to athymic animals is related to tumor cell differentiation and growth kinetics and to host natural killer cell activity. *Invasion Metastasis* 1986; 6:69–82.

13 Linden CJ, Johansson L. Progressive growth of a human pleural mesothelioma xenografted to athymic rats and mice. *Br J Cancer* 1988; 58:614–8.

14 Mauro K, Ueyama Y, Kuwahara Y, *et al*. Human tumour xenografts in athymic rats and their age dependence. *Br J Cancer* 1982; 45:786–9.

15 Stragand JJ, Drewinko B, Henderson SD, *et al*. Growth characteristics of human colonic adenocarcinomas propagated in the Rowett athymic rat. *Cancer Res* 1982; 42:3111–5.

16 Shouval D, Schuger L, Levij IS, *et al*. Comparative morphology and tumourigenicity of human hepatocellular carcinoma cell lines in athymic rat and mice. *Virchows Archiv A, Pathol Anat Histopathol* 1988; 412:595–606.

17 Howard RB, Chu H, Zeligman BE, *et al*. Irradiated nude rat model for orthotopic human lung cancers. *Cancer Res* 1991; 51:3274–80.

18 Kjonniksen I, Nesland JM, Pihl A, Fodstad O. Nude rat model for studying metastasis of human tumor cells to bone and bone marrow. *J Nat Cancer Inst* 1990; 82:408–12.

19 Manusama ER, Durante NM, Marquet RL, Eggermont AM. Ischemia promotes the anti-tumor effect of tumor necrosis factor alpha (TNF α) in isolated limb perfusion in the rat. *Regional Cancer Treat* 1994; 7:155–9.

20 Manusama ER, Nooijen PTGA, Stavast J, *et al*. Synergistic antitumour effect of recombinant human tumour necrosis factor-α with melphalan in isolated limb perfusion in the rat. *Br J Cancer* 1996; 83:551–5.

21 Milas M, Feig B, Yu D, *et al*. Isolated limb perfusion in the sarcoma-bearing rat: a novel preclinical gene delivery system. *Clin Cancer Res* 1998; 3:2197–203.

22 Sheil JM, Gallimore PH, Zimmer SG, Sopori ML. Susceptibility of adenovirus 2-transformed rat cell lines to natural killer (NK) cells: direct correlation between NK resistance and *in vivo* tumorigenesis. *J Immunol* 1984; 132:1578–2.

23 Rauck A, McCart L, Martinez D, Qualman S. A nude rat model of human neuroblastoma using orthotopic implantation (Meeting abstract). *Proc Annu Meet Am Assoc Cancer Res* 1993; 34:A347.
